# Eye-tracking evidence of a relationship between attentional bias for emotional faces and depression severity in patients with treatment-resistant depression

**DOI:** 10.1038/s41598-024-62251-4

**Published:** 2024-05-25

**Authors:** Laetitia Imbert, Cécilia Neige, Rémi Moirand, Giulia Piva, Benoit Bediou, William Vallet, Jerome Brunelin

**Affiliations:** 1 Université Claude Bernard Lyon 1, CNRS, INSERM, Centre de Recherche en Neurosciences de Lyon CRNL U1028 UMR5292, PSYR2, 69500 Bron, France; 2 Centre Hospitalier Le Vinatier, Bat 416 1st floor, 95 boulevard Pinel, 696500 Bron, France; 3 Faculté de Psychologie et Sciences de l’Education Campus Biotech, Université de Genève, 9 chemin des mines, 1211 Geneva, Switzerland

**Keywords:** Major depression, Facial emotion, Free-viewing task, Emotional bias, Emotion, Depression

## Abstract

In a retrospective study, 54 patients with treatment-resistant major depressive disorder (TRD) completed a free-viewing task in which they had to freely explore pairs of faces (an emotional face (happy or sad) opposite to a neutral face). Attentional bias to emotional faces was calculated for early and sustained attention. We observed a significant negative correlation between depression severity as measured by the 10-item Montgomery-Åsberg Depression Rating Scale (MADRS) and sustained attention to happy faces. In addition, we observed a positive correlation between depression severity and sustained attention to sad faces. No significant correlation between depression severity and early attention was found for either happy or sad faces. Although conclusions from the current study are limited by the lack of comparison with a control group, the eye-tracking free-viewing task appears to be a relevant, accessible and easy-to-use tool for measuring depression severity through emotional attentional biases in TRD.

## Introduction

Major depressive disorder (MDD) is one of the most common mental health disorders, affecting 4.4% of the world’s population^1^. In addition to the classic clinical symptoms (e.g., feelings of guilt, sadness), MDD is also characterized by cognitive impairments that contribute to the overall burden of the illness. Among these, deficits in emotional processing have attracted a particular interest because of their hypothesized role in the onset and maintenance of depressive symptoms^2^. Several studies have shown that, compared to healthy controls, people with MDD exhibit a bias toward negative stimuli over positive or neutral information^3^, and conversely, tend to neglect positive information^4^.

Interestingly, significant relationships were observed between depression severity and the intensity of emotional biases. One study using different types of stimuli (e.g., emotional words, scenes, and faces) found an association between depression severity and attention to negative information^5^, while another study using only emotional words found no relationship^6^, calling for further research on this topic.

Better characterizing attentional biases to emotional stimuli in MDD patients is crucial, as they have been shown to be a stable vulnerability factor for depression^2^, to persist in remitted depression and to predict future depressive symptomatology^7^.

It is worth noting that the mentioned studies investigating attentional biases in MDD have primarily used motor reaction time tasks and indirect measures of attention such as the stroop, dot-probe or cueing tasks. However, these tasks primarily assess early attention allocation (direction bias) which is thought to be less impaired in MDD than sustained attention allocation (duration bias)^8,9^*.* In addition, reaction time measures used to assess attentional processes have extremely low reliability^10^ and do not accurately capture emotional processing in patients who have difficulty responding by pressing a keyboard, due to psychomotor retardation or catatonic symptoms. Eye-tracking can overcome these limitations by providing a direct and objective measure of sustained and early attention through the recording of eye movement.

Free-viewing eye-tracking meta-analysis and review have reported that patients with MDD show a robust attentional bias away from positive stimuli and a less robust bias toward negative stimuli, compared to controls^9,11^. Importantly, patients with MDD show a more pronounced attentional bias toward negative stimuli than participants with subclinical depression^11^. Several studies have examined the relationship between depression severity and attentional bias toward emotional faces using eye-tracking in patients with MDD, but have yielded inconsistent conclusions. Some have focused solely on investigating the relationship between symptom severity and sustained attentional bias for both positive and negative information^12–14^ revealing no significant correlation. Conversely, other studies have used a free-viewing task to examine both early and sustained attention, leading to conflicting results. For instance, Duque and Vázquez^15^ identified a significant correlation only between the depression severity and the sustained attentional bias for sad faces, whereas Bodenschatz et al.^16^ reported no such correlation.

To address these discrepancies presumed to stem from methodological differences, the present study aims to investigate the relationship between both sustained and early attentional biases for emotional faces (happy and sad) and depression severity in patients with moderate to severe treatment-resistant MDD (TRD) with stable medication for 4 weeks. In this study, we hypothesized that there would be inverse correlations between attentional bias to positive and negative emotions and the severity of depression. The ultimate goal of this study is to establish eye tracking in a free-viewing task as a reliable tool for assessing the severity of depression.

## Results

### Clinical and sociodemographic characteristics

Clinical and sociodemographic characteristics are detailed in Table 1.

**Table 1:** Clinical and sociodemographic characteristics of the population included in the analysis.

	n = 54
Age (Mean, SD, [min–max])	49.57 ± 9.73 [24–63]
Male/Female	22/32
MADRS (Mean, SD, [min–max])	29.00 ± 6.19 [20–49]
Maudsley score (Mean, SD, [min–max])	8.41 ± 1.81 [5–13]
Antidepressant medication (n)	
SSRIs	24
SNRIs	17
TCAs	18
Other (MAOI + agonist DA)	5
Other medication (n)	
BZD	20
Antipsychotics	15

Results are given as mean (± standard deviation).

MADRS, montgomery and asberg depression rating scale; SSRI, selective serotonin reuptake inhibitor; SNRI, serotonin and noradrenaline reuptake inhibitors; TCA, tri/tetracyclic antidepressant; MAOI, monoamine oxidase inhibitor; DA, dopamine; BZD, benzodiazepine.

### Relationship between depression severity and sustained emotional attentional bias

**Figure 1:**
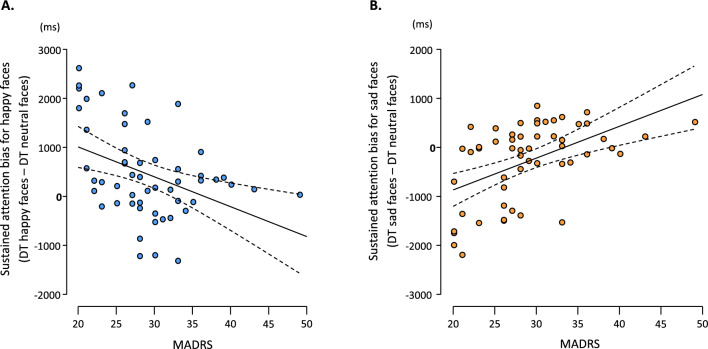
Correlation between depression severity and emotional bias for sustained attention. (**A**) Significant negative correlation between depression severity and sustained attention bias for happy faces (r = − 0.40; p_corr_ < 0.01; BF_10_ = 14.77). (**B**) Significant positive correlation between depression severity and sustained attention bias for sad faces (r = 0.51; p_corr_ < 0.001; BF_10_ = 281.8).

A significant negative correlation between depression severity and sustained attention bias for happy faces (*p*_corr_ < 0.01; *r* = -0.40; IC_95%_ = [− 0.61; − 0.15]) was observed with strong evidence in favor of H_1_ (BF_10_ = 14.77; Fig. 1A). There was also a significant positive correlation between depression severity and sustained attention bias for sad faces (*p*_corr_ < 0.001; *r* = 0.51; IC_95%_ = [0.28; 0.68]) with decisive evidence in favor of H_1_ (BF_10_ = 281.8; Fig. 1B). Multiple linear regression analyses showed no effect of age or sex on sustained attentional bias (see Table 2).

**Table 2:** Multiple linear regression analyses of the MADRS score, age, and sex on sustained attentional bias to happy faces and sustained attentional bias to sad faces.

Model	Unstandardized coefficients		Standardized coefficients	*t*	*p*
	B	Std. error	Beta		
HAPPY					
(Constant)	1560.95	735.17		2.123	0.039
MADRS	− 66.48	19.57	− 0.440	− 3.398	0.001**
Sex	133.25	258.63	0.071	0.515	0.609
Age	11.99	10.90	0.150	1.101	0.276
SAD					
(Constant)	− 1923.35	596.33		− 3.225	0.002
MADRS	67.79	15.87	0.528	4.271	< 0.001**
Sex	− 153.09	209.79	− 0.096	− 0.730	0.469
Age	− 2.09	8.84	− 0.031	− 0.237	0.814

Significant values are in [italics].

For sustained attentional bias toward happy faces, the multiple linear regression model accounted for 19.7% of the variation in the attentional bias to happy faces and reached statistical significance (*F*_(3,50)_ = 4.088, p = 0.011; R = 0.444; adj. R square = 0.149). The results show that only the MADRS score was statistically significant in predicting the attentional bias to happy faces while sex and age were not. For sustained attentional bias to sad face, the overall model explained 26.8% of the variation in the attentional bias to sad faces and was significant (*F*_(3,50)_ = 6.093, p = 0.001; R = 0.517; adj. R square = 0.224). Again, the results show that only the MADRS score was statistically significant in predicting the attentional bias to sad faces while sex and age were not (Table 2).

### Relationship between depression severity and early emotional attentional bias

Analyses revealed no significant correlation between the depression severity and the laterality quotient for either happy faces (*p*_corr_ = 0.052; *r* = 0.34; IC_95%_ = [0.08; 0.56]; substantial evidence in favor of H_1_: BF_10_ = 6.846, n = 53) or sad faces (*p*_corr_ = 0.90; *r* = -0.17; IC_95%_ = [− 0.11; 0.42]; anecdotal evidence in favor of H_0_: BF_10_ = 0.35).

## Discussion

We aimed to characterize the relationship between depression severity and emotional bias using a straightforward measure of sustained and early attention in patients with TRD.

Regarding sustained attention, the analyses showed strong evidence for a negative correlation between emotional bias to happy faces and the depression severity, and decisive evidence for a positive correlation between emotional bias to sad faces and the depression severity. This suggests that the more severe the depression, the more time participants spent looking at sad faces and the less time they spent looking at happy faces compared to neutral faces. Strengthening this robust association between depression severity and attentional bias, multiple linear regression analyses show that only the MADRS score is a predictor of sustained attentional bias, while gender and age do not contribute significantly to the prediction. Some of these findings are supported by previous eye-tracking studies, which have reported a more pronounced attentional bias in patients diagnosed with MDD compare to participants with subclinical depression^11^, a positive correlation between depression severity and sustained attentional bias to sad faces^15^, a longer DTs to sad faces, and a lack of bias to happy faces compared to controls^17,18^.

Regarding early attentional biases, no significant relationships were observed between the orientation of the first saccade to sad and happy faces and the depression severity. These findings are consistent with several previous eye-tracking studies showing that people with MDD do not show an initial orientation bias to emotional stimuli^8,9^.

The current study replicates and extends previous findings by showing in the same FVT study that there is an association between sustained emotional bias for both positive and negative stimuli and depression severity, but not with early emotional attentional bias. Although these results are encouraging some limitations that curtail the generalization of the results must be noted. First, our statistical model only controls for the effect of MADRS score, age and sex on cognitive bias, but it is known that other variables could have an impact on this cognitive function, such as treatments or disease characteristics (course of the disease, duration of episode, number of relapses, etc.). Second, it should be noted that results were obtained in patients with TRD, a specific subgroup of patients that may present differences from a clinical^19^ and neurobiological point of view^20^. However, the inclusion of treatment-resistant patients with stable antidepressant treatment for at least 4 weeks allowed us to control for the potential beneficial effect of medication on emotional processing, including FVT^21^. Third, the study suffers from lack of a comparison group (either healthy volunteers or people with subclinical depression). Finally, the number of face pairs presented may seem small, but this allows us to propose a task that can be completed quickly and integrated into the patient's examination routine (< 5 min). Then, because we only included ‘sad’ and ‘happy’ faces, the generalization of the current conclusions to other emotions is limited.

Finally, using a task that is accessible and easy to integrate into patients' assessment routines, we showed that sustained attentional bias toward sad and away from happy faces increases with depression severity. The FVT may be a useful tool for objective assessment of depression severity in clinical settings.

## Material and method

This retrospective study was approved by a local ethics committee (Comité d’Ethique de la REcherche du VInatier—CEREVI, id number #2023/006, on April 24th, 2023), was performed in accordance with relevant French guidelines and regulations using data from an authorized anonymized database (Commission Nationale de l’Informatique et des Libertés CNIL, MR-003-2017-002), and in accordance with the Declaration of Helsinki. All participants (and/or their legal guardians when applicable) gave written informed consent for participation and for publication of the study. This retrospective study was not registered in a public database prior to its execution.

### Sample

We reviewed data from patients who attended our clinical unit for treatment-resistant depression between December 2018 and December 2022. Treatment-resistant depression was defined as a failure to respond to two or more antidepressant regimens despite adequate dose and duration and adherence to treatment^22^. The 10-item Montgomery-Asberg Depression Rating Scale (MADRS_10_)^23^ was used to assess depression severity. It is a clinician-rated questionnaire known for its validity and inter-rater reliability^24^. It is designed to assess depression severity through 10 items that focuses on the symptoms of depression (e.g., sadness, tension, and pessimistic thoughts).

Patients were eligible if they had a DSM-5 diagnosis of MDD, have moderate to severe depression as indicated by a MADRS_10_ score ≥ 20 even on stable medication (dosage and molecule) for 4 weeks, and have completed the free viewing task (FVT) on the day of their first visit to our clinical unit. Exclusion criteria included neurological (e.g., dementia) or psychiatric comorbidities, unanalyzable eye-tracking data, incomplete clinical information, or withdrawal of consent. Of the 61 eligible patients, 7 patients were not included due to unprocessable eye-tracking data, resulting in a final analyzed sample of 54 participants with moderate to severe depression.

### Eye tracking free-viewing task

The FVT consisted of showing pairs of faces: one emotional (happy or sad) and one neutral, presented randomly for 3500 ms to the right and left of a fixation cross lasting 1500 ms. We presented 23 pairs of stimuli: 11 happy-neutral and 12 sad-neutral pairs, with 12 different identities (6 males, 6 females). The faces were extracted from the Ekman and Friesen open set^25^. Throughout the task, gaze position and eye movements were recorded with an eye-tracking system (SMI SensoMotoric Instruments with BeGaze 3.6.52, Teltow, Germany).

### Eye tracking measurement analysis

Early (direction bias) and sustained (duration bias) attention allocation was assessed per participant based on eye-movements recording^26^.

Dwell time (DT), the total time (ms) of all fixations on the faces, was used to measure sustained attention allocation (duration bias). The difference between emotional and neutral DTs was used to calculate an emotional bias score.

The orientation of the first saccade, corresponding to the first face the participant looked at, was used to measure early attention allocation (direction bias). A laterality quotient was calculated as the difference between the number of times the participant made a first saccade to the emotional face and the number of times where the first saccades were to the neutral face, divided by the total number of trials.

For both measures, a positive value indicates a bias toward emotional faces, while a negative value indicates a bias toward neutral faces.

### Statistical analysis

We used Pearson’s correlations with Bonferroni adjustment (*p*_corr_) to examine the relationship between sustained and early emotional attentional biases and depression severity (MADRS_10_ scores). To complement the frequentist statistics, we also performed Bayesian analyses using BF_10_, which provided us with a likelihood ratio of the alternative hypothesis (i.e., H_1_: correlation between variables) to the null hypothesis (i.e., H_0_: no correlation). Finally, to provide a more comprehensive understanding of the findings, multiple linear regression analyses were conducted to assess the predictive significance of the MADRS score, age, and sex on sustained attentional bias to happy faces and sustained attentional bias to sad faces. All statistical analyses were performed using Rstudio version 2021.09.2+382 (Richmond Hill, Canada).

## Data Availability

The data that support the findings of this study are available on request from the corresponding author, [J.B.]. The data are not publicly available due to local restrictions (e.g., their containing information that could compromise the privacy of research participants).
